# Navigating Nutritional Strategies: A Comprehensive Review of Early and Delayed Enteral Feeding in Acute Pancreatitis

**DOI:** 10.7759/cureus.53970

**Published:** 2024-02-10

**Authors:** Suprit Malali, Shilpa A Gaidhane, Sourya Acharya, Harshitha Reddy, Nikhil Pantbalekundri

**Affiliations:** 1 Medicine, Jawaharlal Nehru Medical College, Datta Meghe Institute of Higher Education & Research, Wardha, IND

**Keywords:** severity grading, nutritional strategies, delayed initiation, early initiation, enteral feeding, acute pancreatitis

## Abstract

This review critically examines enteral feeding strategies in managing acute pancreatitis, focusing on the contrasting early and delayed initiation approaches. Acute pancreatitis, marked by pancreatic inflammation, poses complex challenges, and nutritional interventions are pivotal in patient outcomes. Early enteral feeding, initiated within 24-48 hours, is associated with positive outcomes such as shortened hospital stays and reduced complications. However, controversies persist, with studies questioning its universal benefits. Conversely, delayed enteral feeding, employing a cautious approach, gains prominence in high-risk and severe cases. The identification of high-risk patients becomes paramount in decision-making. Practical recommendations for clinicians advocate an individualized approach, considering the severity of pancreatitis and regular monitoring. As the landscape of acute pancreatitis management evolves, staying abreast of emerging guidelines is essential. This review aims to provide a comprehensive understanding of critical findings, offering practical insights to guide clinicians in navigating the complexities of enteral feeding decisions in acute pancreatitis.

## Introduction and background

Acute pancreatitis, characterized by the inflammation of the pancreas, is a condition with diverse etiologies ranging from gallstones and alcohol consumption to metabolic disorders. The pathophysiology involves the activation of pancreatic enzymes within the gland, leading to autodigestion and tissue damage. This inflammatory cascade affects the pancreas and triggers a systemic response, impacting various organs and systems. Amidst the multifaceted challenges posed by acute pancreatitis, the role of nutritional strategies emerges as a critical aspect in managing this complex condition [1]. Acute pancreatitis is defined by the sudden onset of inflammation in the pancreas, characterized by abdominal pain, elevated pancreatic enzymes, and, in severe cases, systemic complications. The severity of the condition can range from mild, self-limiting cases to severe forms associated with necrosis, organ failure, and high mortality rates [1].

The significance of nutritional interventions in acute pancreatitis extends beyond traditional supportive care. The inflammatory response and gastrointestinal dysfunction accompanying the condition often led to malnutrition, exacerbating the patient's health. Nutritional strategies play a pivotal role in preventing and addressing malnutrition, modulating the inflammatory response, supporting organ function, and influencing disease course [2].

Controversies surrounding the optimal timing and approach to enteral feeding in acute pancreatitis have been a subject of ongoing debate within the medical community. This review aims to critically examine the existing literature, providing insights into the controversies of enteral feeding practices. By evaluating the strengths and limitations of different studies, we aim to contribute to the ongoing discourse on the most effective and evidence-based approaches to enteral feeding in acute pancreatitis. One of the central debates in managing acute pancreatitis revolves around the timing of enteral feeding initiation. Some advocate for early enteral feeding (EEF) to preserve nutritional status and prevent complications, while others argue in favor of delayed initiation, emphasizing the potential risks associated with feeding in the acute phase. This review will delve into the available evidence, comparing the outcomes of EEF and delayed enteral feeding (DEF) and exploring the factors that influence the decision-making process for clinicians.

## Review

EEF in acute pancreatitis

Definition and Rationale

EEF denotes the commencement of nutritional support via the gastrointestinal tract during the initial stages of acute pancreatitis, typically within the initial 24-48 hours following admission. The justification for EEF is rooted in its capacity to alleviate the adverse impacts of malnutrition and mitigate the systemic inflammatory response linked to acute pancreatitis. This methodology seeks to uphold gastrointestinal integrity, regulate the immune response, and forestall complications arising from extended periods of fasting [3].

Clinical Studies Supporting EEF

Positive outcomes: Numerous clinical studies and meta-analyses substantiate the favorable outcomes associated with EEF in the context of acute pancreatitis. A comprehensive meta-analysis involving 11 studies encompassing 775 patients concluded that EEF significantly correlated with reduced rates of infections [3]. Another meta-analysis, comprising six studies, underscored that EEF initiated within 48 hours, as opposed to DEF, significantly decreased the risks of multiple organ failure [4]. Additionally, an encompassing meta-analysis highlighted that EEF within the initial 48 hours of admission exhibited enhanced clinical outcomes for acute pancreatitis by mitigating complications [5]. Moreover, an examination of published studies about pediatric acute pancreatitis concurred, indicating a positive association between EEF and improved clinical outcomes [6]. Collectively, these findings robustly support the potential benefits of EEF in managing acute pancreatitis.

Reduction in complications: Many clinical studies further corroborate the advantageous impact of EEF on reducing complications in acute pancreatitis. A prospective non-randomized controlled study explicitly targeting patients with acute moderate and severe pancreatitis concluded that early enteral nutrition (EEN) was both safe and beneficial, reporting a discernibly lower incidence of complications in the EEF group when compared to the DEF group [7]. A systematic review, comprehensively analyzing current data on enteral nutrition in acute pancreatitis, identified the potential benefits of an early low-fat oral diet, particularly in cases of mild pancreatitis. The review also underscored the comparable safety profiles of nasogastric and nasojejunal routes [3]. A randomized controlled trial focusing on pediatric acute pancreatitis patients demonstrated that the implementation of EEN, coupled with aggressive fluid resuscitation, significantly contributed to improved clinical outcomes [6]. A Preferred Reporting Items for Systematic Reviews and Meta-Analyses (PRISMA)-compliant systematic review and meta-analysis comparing EEN with delayed enteral nutrition (DEN) in acute pancreatitis indicated a lower incidence of complications associated with EEN [8]. These studies provide compelling evidence supporting the assertion that EEF in acute pancreatitis plays a pivotal role in reducing complications and ultimately enhancing patient outcomes.

Considerations for Patient Selection

Severity of pancreatitis: EEF in acute pancreatitis has demonstrated notable benefits, particularly in reducing complications. However, the critical consideration of patient selection based on the severity of pancreatitis is imperative. Studies indicate that EEF yields more significant advantages, including shortened hospital stays and decreased pain scores, in patients with acute moderate and severe pancreatitis [7]. While a prospective non-randomized controlled study affirmed the safety and benefits of EEN in this subgroup, acknowledging fewer complications in the EEF group compared to DEN [7], the study's design raises concerns about potential selection bias, given the non-random assignment of patients to EEF or DEF [7]. Current evidence underscores the benefits of EEN in severe pancreatitis, emphasizing the equivalence of gastric and jejunal feeding efficacy. The decision to initiate EEF should be tailored to individual cases, considering the patient's overall condition and specific risk factors for complications. In essence, while EEF holds promise in reducing complications in acute pancreatitis, judicious patient selection based on the severity of the condition remains paramount to ensure appropriateness and safety for each individual.

Timing of initiation: The timing of EEF initiation in acute pancreatitis emerges as a crucial factor in patient selection. Existing evidence advocates for the benefits of EEF in severe pancreatitis, recommending initiation within the initial 24 hours of diagnosis [9,10]. A meta-analysis of randomized controlled trials further supports the notion that immediate enteral nutrition can expedite recovery and is safe, particularly in cases of mild acute pancreatitis [4]. However, the decision to initiate EEF should be nuanced, considering the patient's overall condition and specific risk factors for complications. A prospective non-randomized controlled study affirms the safety and benefits of EEN in patients with acute moderate and severe pancreatitis, observing fewer complications in the EEF group compared to the DEF group [7]. Additionally, a study reports that patients receiving oral or enteral feeds within 24 hours of acute pancreatitis diagnosis are less likely to experience prolonged hospital stays [10]. In essence, while current evidence supports the benefits of EEN in severe pancreatitis and recommends initiation within the first 24 hours, initiating EEF should be individualized, considering the patient's overall condition and specific risk factors for complications.

DEF in acute pancreatitis

Definition and Rationale

DEF in acute pancreatitis entails postponing the commencement of nutritional support beyond the initial stages of the disease, typically deferring until the inflammatory response has subsided. The underlying rationale for adopting DEF revolves around apprehensions regarding the possible exacerbation of inflammation and complications associated with early nutritional support. This approach is strategically designed to mitigate the potential risks of complications, especially in patients confronting severe pancreatitis or those deemed at elevated risk for adverse outcomes [3]. By intentionally delaying enteral feeding until a more judicious juncture in the disease course, clinicians aim to strike a balance that minimizes potential harm and optimizes the overall management of acute pancreatitis.

Contrasting Studies and Controversies

Studies questioning early initiation: Adopting DEF in managing acute pancreatitis has spurred considerable investigation and debate. A prospective non-randomized controlled study, while affirming the safety and benefits of EEN in patients with acute moderate and severe pancreatitis, reported fewer complications in the EEF group compared to the DEF group [7]. However, the study's design raises concerns about potential selection bias, given the non-random assignment of patients to receive EEF or DEF [7]. A systematic review analyzing current data on enteral nutrition in acute pancreatitis identified potential benefits of an early low-fat oral diet, particularly in mild cases. It underscored the comparable safety profiles of the nasogastric and nasojejunal routes [3]. Contrarily, a randomized controlled trial focusing on pediatric acute pancreatitis patients revealed improved clinical outcomes with EEN and aggressive fluid resuscitation [6]. A PRISMA-compliant systematic review and meta-analysis comparing EEN and DEN in acute pancreatitis demonstrated fewer complications associated with EEN [6]. Despite initial promising results, current evidence does not support using immuno-enhanced nutrients or probiotics in acute pancreatitis [3]. The timing of enteral nutrition initiation remains contentious, necessitating further research to establish clear guidelines for patient selection and timing in acute pancreatitis.

Identification of high-risk patients: Various studies and reviews have shed light on using enteral nutrition in acute pancreatitis, explicitly focusing on identifying high-risk patients and the optimal timing for initiation. A systematic review and meta-analysis advocated for the early commencement of enteral feeding within the first 24 hours, with the safety of the nasogastric route comparable to that of the nasojejunal route [3]. Another meta-analysis of randomized controlled trials affirmed that enteral nutrition within 48 hours of admission improves clinical outcomes and reduces complications in acute pancreatitis [5]. In identifying high-risk patients, a prospective non-randomized controlled study reported that EEN, particularly in acute moderate and severe pancreatitis, resulted in fewer complications than DEN [7]. The concept of "pancreatic rest" lacks evidence-based support, emphasizing the importance of EEN, particularly in moderately severe or severe pancreatitis, to maintain gut function and achieve positive clinical outcomes [9]. These findings underscore the potential benefits of EEF, initiated within the first 24-48 hours of admission, in reducing complications and improving clinical outcomes in acute pancreatitis. Additionally, the severity of the condition should be carefully considered when identifying high-risk patients, as EEN emerges as particularly advantageous in acute moderate and severe pancreatitis cases.

Factors Influencing DEF

Presence of complications: The decision regarding DEF in acute pancreatitis is intricately linked to various factors, with complications being a critical consideration. Numerous studies have investigated the impact of enteral feeding timing on complications and patient outcomes. A randomized controlled trial conducted at Victoria Hospital, Bengaluru, India, specifically comparing EEF and DEF in severe acute pancreatitis cases revealed that EEF correlated with a shorter time to achieve complete tolerance to enteral feeds, reduced admission duration, and fewer complications such as necrotizing pancreatitis and organ failure, ultimately resulting in lower mortality rates when compared to DEF [11]. Further support for early initiation comes from a meta-analysis published in PLOS One, which concluded that enteral nutrition initiated within 48 hours of admission significantly improved clinical outcomes in acute pancreatitis by effectively reducing complications [5]. These findings underscore the pivotal role of enteral feeding timing, particularly early initiation, in mitigating complications and enhancing overall outcomes in acute pancreatitis. Thus, complications should be paramount when determining the optimal timing for enteral feeding initiation in patients with acute pancreatitis.

Severity grading: The severity grading of acute pancreatitis emerges as a crucial determinant influencing the decision to initiate DEF. Multiple factors associated with the severity of the condition can impact the timing of enteral feeding initiation. Notably, a study highlighted that EEF correlated with significantly reduced pain scores, shorter hospital stays, and fewer nutrition-related complications in acute pancreatitis patients [7,10]. Complications such as multiple organ failure and necrotizing pancreatitis, often indicative of higher severity, play a pivotal role in guiding the decision to initiate DEN. The evidence consistently supports the notion that EEF reduces the risk of these complications [7,10]. Moreover, EEF has demonstrated superiority in fewer nutrition-related complications and significantly reduced mortality rates compared to parenteral feeding [3]. In considering the severity grading of acute pancreatitis, factors encompassing pain severity, hospital stay duration, the presence of complications, nutrition-related complications, and mortality rates should collectively inform the decision-making process regarding the timing of enteral feeding initiation. In doing so, clinicians can tailor their approach to provide optimal care for patients with acute pancreatitis.

Comparative analysis

Outcomes of EEF Versus DEF

Mortality rates: Examining mortality rates in EEF versus DEF in acute pancreatitis draws insights from diverse studies. Comparative analyses often distinguish between EEN, initiated within 48 hours of admission, and DEN, initiated after 48 hours. Findings indicate a need for more apparent differences in the 30-day mortality rates between the two groups [12]. Nevertheless, certain studies suggest a trend favoring reduced mortality associated with EEN [13]. A randomized controlled trial conducted at Victoria Hospital, Bengaluru, reported that EEF was linked to a shorter duration for complete tolerance to enteral feeds, fewer admission days, and a diminished incidence of complications, including necrotizing pancreatitis and organ failure, ultimately translating into reduced mortality compared to DEF [11]. A systematic review and a subsequent PRISMA-compliant systematic review with PRISMA extension concurred that EEN correlated with fewer complications and improved clinical outcomes in acute pancreatitis [14]. Despite the absence of a clear consensus on the impact of EEF versus DEF on mortality rates in acute pancreatitis, several studies hint at a potential trend toward reduced mortality with EEN. However, further research is imperative to establish a definitive conclusion regarding the optimal timing of enteral feeding in acute pancreatitis.

Complication rates: The comparative analysis of EEF versus DEF in acute pancreatitis, explicitly on complication rates, reveals a nuanced landscape with varied study outcomes. While some studies report a reduction in complications associated with EEN, others indicate no significant difference between EEN and DEN. A retrospective cohort study exploring EEF versus DEF in patients with abdominal trauma found that the early-initiation group experienced fewer infectious complications and shorter lengths of stay in both the surgical intensive care unit (ICU) and the hospital compared to the delayed-initiation group [15]. In alignment with these findings, a systematic review and meta-analysis of randomized controlled trials concurred that EEN was linked to fewer complications and improved clinical outcomes in acute pancreatitis [12]. The randomized controlled trial at Victoria Hospital, Bengaluru, reinforced this trend, reporting fewer complications, including necrotizing pancreatitis and organ failure, and reduced mortality with EEF compared to DEF [11]. However, a Cochrane review yielded no apparent differences in 30-day mortality rates or intolerance to feeding between the EEF and DEF groups [12]. The mixed results from studies comparing EEF and DEF in acute pancreatitis underscore the need for further research to establish a definitive conclusion regarding the optimal timing of enteral feeding in this clinical context.

Patient-Centered Considerations

Tolerance and compliance: Patient-centered considerations, explicitly focusing on tolerance and compliance, are pivotal in enhancing health outcomes. Extensive research underscores the correlation between patient-centered care and improved adherence, leading to better health outcomes [16]. Patient-centeredness in healthcare is associated with heightened patient engagement, satisfaction, and compliance, ultimately contributing to an elevated quality of life (QoL) and diminished patient anxiety [17]. Furthermore, aligning care with patient preferences and values has been linked to enhanced compliance, patient satisfaction, and overall improved recovery and health outcomes [18]. Overcoming barriers to patient adherence is deemed crucial, and integrating innovative drug delivery systems is recognized as a valuable strategy to enhance patient adherence [19]. A patient-centered approach necessitates identifying and negotiating diverse communication styles, decision-making preferences, and the inclusion of family roles, thereby fostering cultural competence in healthcare delivery [20].

QoL: In managing acute pancreatitis, patient-centered considerations, particularly about QoL, emerge as indispensable. EEN has been associated with improved clinical outcomes, including enhanced QoL, in individuals with acute pancreatitis [3]. The findings from a randomized controlled trial conducted at Victoria Hospital, Bengaluru, underscored that EEF correlated with fewer complications, encompassing necrotizing pancreatitis and organ failure, and reduced mortality compared to DEF [9]. A systematic review and meta-analysis of randomized controlled trials further affirmed that EEN correlated with fewer complications and improved clinical outcomes in acute pancreatitis [3]. Emphasizing patient-centered care, which actively acknowledges and integrates patients' values, has been correlated with heightened engagement, satisfaction, and compliance, thereby contributing to improved QoL and decreased patient anxiety [11]. Consequently, considering QoL emerges as a central tenet of patient-centered care, ensuring that healthcare delivery aligns with patients' goals and expectations. The active involvement of patients in clinical decision-making processes has been shown to enhance outcomes, including QoL, highlighting the essential role of patient-centered care in improving health outcomes for individuals dealing with chronic illnesses.

Nutritional support strategies

Types of Enteral Nutrition

Elemental versus polymeric formulas in acute pancreatitis: Enteral nutrition is a crucial component in managing acute pancreatitis, with elemental and polymeric formulas representing two common types of enteral nutrition. Elemental formulas contain predigested nutrients, facilitating easier absorption, and are typically recommended for patients with malabsorption issues or intolerance to polymeric formulas. On the other hand, polymeric formulas consist of whole proteins and are suitable for patients with normal gastrointestinal function. A systematic review and meta-analysis of randomized controlled trials underscored that EEN correlated with fewer complications and improved clinical outcomes in acute pancreatitis [7]. In parallel, findings from a randomized controlled trial at Victoria Hospital, Bengaluru, highlighted that EEF was linked to reduced complications, including necrotizing pancreatitis, organ failure, and lower mortality, than DEF [6]. The patient's needs and tolerance should guide the selection between elemental and polymeric formulas. Current evidence does not support using immuno-enhanced nutrients or probiotics in acute pancreatitis [3]. Thus, the choice of enteral nutrition formulas should be based on individual patient considerations, emphasizing the importance of tailoring nutritional support to meet specific needs and tolerances while avoiding immuno-enhanced nutrients or probiotics based on the current lack of supporting evidence.

Monitoring and Adjustments

Nutritional assessment in acute pancreatitis: In the comprehensive management of acute pancreatitis, continuous monitoring and adjustments of nutritional support strategies are paramount. Central to this process is nutritional assessment, a vital component involving the evaluation of the patient's nutritional status, encompassing parameters such as body weight, body mass index, and serum albumin levels [3]. Evidence from a meta-analysis of randomized controlled trials reinforces the significance of EEN within the first 48 hours of admission, revealing its association with improved clinical outcomes in acute pancreatitis [4]. Correspondingly, a randomized controlled trial at Victoria Hospital, Bengaluru, affirms that EEF is linked to reduced complications, including necrotizing pancreatitis, organ failure, and lowered mortality, compared to DEF [6]. The selection of the enteral nutrition formula should be a tailored decision, considering the patient's needs and tolerance. Notably, immuno-enhanced nutrients or probiotics lack support from current evidence [3]. Hence, selecting enteral nutrition formulas should be individualized, aligning with the patient's needs and tolerance while avoiding using immuno-enhanced nutrients or probiotics based on the current lack of supporting evidence.

Biomarkers and imaging in acute pancreatitis: Biomarkers and imaging emerge as indispensable tools in the multifaceted management of acute pancreatitis. Imaging biomarkers, identifiable features in images relevant to diagnosis, staging, or monitoring, are pivotal in assessing treatment responses, especially in genetically diverse patient subgroups presenting with similar symptoms [21,22]. Biomarkers, biological molecules in the blood, other body fluids, or tissues, indicate normal or abnormal processes, conditions, or diseases [23]. In acute pancreatitis, imaging biomarkers prove invaluable for swiftly assessing treatment responses in genetically distinct patient subsets with convergent symptoms [22]. Furthermore, these biomarkers aid in uncovering the underlying mechanisms of the disease, paving the way for identifying potential therapeutic targets [23]. Rigorous validation and qualification are imperative for incorporating biomarkers into research or healthcare settings [24]. Notably, developing and evaluating cancer biomarkers, focusing on biospecimen-derived biomarkers, have been explored extensively [24]. Advancing quantitative imaging biomarkers as decision-support tools in clinical practice necessitates harmonizing data acquisition and analysis [25]. The advantages of quantitative imaging biomarkers over biomaterials include automated quantitation, disease recognition thresholds, and the ease of transfer for biomarker extraction in an automated, reproducible, and blinded manner [25]. In summary, biomarkers and imaging are integral components in the dynamic management of acute pancreatitis, offering insights into treatment responses and contributing to advancements in precision medicine.

Guidelines and recommendations

International Guidelines

American Pancreatic Association (APA) guidelines: International guidelines, particularly those established by the APA and the International Association of Pancreatology (IAP), stand as pivotal resources guiding the effective management of acute pancreatitis. The 2012 IAP/APA guidelines are noteworthy for providing evidence-based recommendations encompassing medical and surgical aspects of acute pancreatitis management [26]. Covering critical areas such as diagnosis, prognostication, imaging, fluid therapy, intensive care management, prevention of infectious complications, nutritional support, and biliary tract management, these comprehensive guidelines serve as a roadmap for clinicians [26]. Key recommendations include cautioning against routine prophylactic antibiotics in severe acute pancreatitis or sterile necrosis cases while acknowledging their potential role in delaying intervention for patients with infected necrosis [27]. The guidelines recommend immediate oral feeding for mild cases without nausea and vomiting. At the same time, enteral nutrition is advised for severe cases to prevent infectious complications, with a preference for avoiding parenteral nutrition [27].

European Society for Clinical Nutrition and Metabolism (ESPEN) guidelines: The ESPEN has issued expansive guidelines addressing clinical nutrition in various healthcare contexts, notably within the ICU and among cancer patients [28-30]. These guidelines are rooted in evidence-based recommendations and offer pragmatic counsel for healthcare practitioners navigating the nutritional care landscape [28-30]. The guidelines include diagnostic criteria for malnutrition, identifying at-risk patients, and enabling targeted interventions to enhance their nutritional status [30]. The guidelines emphasize regular nutritional assessment, especially for those vulnerable to malnutrition, and advocate a proactive approach to monitoring and managing patients' nutritional needs [30]. Personalized nutritional intervention plans, spanning oral, enteral, or parenteral nutrition, are recommended, reflecting a nuanced and patient-centered approach to nutritional care [30]. The guidelines extend their purview to ongoing monitoring and evaluation of nutritional interventions, specifying assessment frequency and appropriate evaluation tools [30]. Recognizing the evolving healthcare landscape, the guidelines underscore the importance of education and training for healthcare professionals managing patients' nutritional status [30]. Addressing key components, the ESPEN guidelines not only provide evidence-based recommendations but also offer practical guidance for healthcare providers navigating the complexities of nutritional management in diverse clinical settings, encompassing the challenges of the ICU and unique considerations for cancer patients [28-30]. The guidelines advocate for a holistic and individualized approach, highlighting regular assessments, personalized interventions, and vigilant monitoring to optimize patients' nutritional well-being [28-30].

Evolving recommendations 

Updates in Recent Guidelines

APA and IAP guidelines: The 2012 IAP/APA guidelines serve as a cornerstone in providing comprehensive recommendations for the medical and surgical management of acute pancreatitis, grounded in the latest available evidence [31]. Continuously evolving, these guidelines undergo updates to incorporate emerging research findings, ensuring that clinicians have access to the most current and relevant information. These guidelines are pivotal in enhancing clinical outcomes by addressing critical aspects of acute pancreatitis management, including diagnosis, prognostication, imaging, fluid therapy, and nutritional support. The commitment to regular updates underscores the dynamic nature of medical knowledge, reflecting a dedication to continuous improvement in patient care.

ESPEN guidelines: ESPEN's guidelines on clinical nutrition are expansive, covering diverse healthcare settings such as the ICU and addressing specific patient populations like those with cancer [32]. These guidelines are not static but adaptive, incorporating new sections to reflect emerging clinical data. Notably, including sections on agents like abatacept and infliximab, assessing their role in treating coronavirus disease 2019 (COVID-19), demonstrates ESPEN's commitment to staying abreast of the latest developments in healthcare. Additionally, the global survey on the Global Leadership Initiative on Malnutrition (GLIM) criteria underscores ESPEN's dedication to evaluating the impact of established criteria on malnutrition management. By continually updating and expanding its guidelines, ESPEN ensures that healthcare professionals access relevant and timely information to optimize nutritional care in various clinical contexts.

COVID-19 treatment guidelines: In the face of the evolving landscape of the COVID-19 pandemic, the COVID-19 Treatment Guidelines Panel operates with agility, providing regular updates to disseminate the most recent information on optimal management [31]. These updates are crucial for healthcare providers and policymakers, offering insights into new recommendations and interventions. Notably, incorporating information on ritonavir-boosted nirmatrelvir (Paxlovid) reflects the commitment to swiftly integrating emerging evidence into treatment guidelines. The dynamic nature of these updates reflects a responsive approach to the rapidly changing understanding of COVID-19, ensuring that healthcare professionals are equipped with the latest knowledge to enhance patient care.

UpToDate: Serving as a dynamic and clinically relevant resource, UpToDate plays a pivotal role in highlighting specific new recommendations and updates that have the potential to alter standard clinical practice [33]. It serves as a bridge between research findings, expert opinions, and evolving medical practices by providing curated information on various medical topics, including acute pancreatitis and nutritional support. Incorporating recent updates in guidelines for acute pancreatitis and nutritional support reflects the platform's commitment to offering healthcare providers the most current and evidence-based information. These updates are essential for clinicians seeking to align their practices with the latest advancements, ultimately contributing to improved patient outcomes and a higher standard of care.

Areas of Ongoing Research

Patient-centered care: Ongoing research endeavors are concentrated on unraveling the intricacies of patient-centered care by identifying and navigating diverse elements such as communication styles, decision-making preferences, the role of family, and considerations of sexual and cultural preferences [20]. The paramount goal is to refine and enhance patient-centered care practices, fostering improved patient engagement, heightened satisfaction levels, and enhanced compliance. This holistic approach not only contributes to elevating the QoL for patients but also catalyzes alleviating patient anxiety, affirming the pivotal role of ongoing research in reshaping healthcare delivery to be more attuned to individual patient needs.

Imaging biomarkers: Contemporary research initiatives are dedicated to advancing the field of imaging biomarkers, emphasizing their development and validation for acute pancreatitis in terms of diagnosis, staging, and ongoing monitoring [22]. Recognizing the potential of imaging biomarkers as a critical tool for swiftly assessing treatment responses, particularly in genetically diverse patient subgroups with analogous symptoms, underscores the transformative impact of ongoing research in this domain. The developmental journey of new imaging biomarkers involves meticulous calibration and quality control measures for machinery, equipment, and software, alongside the imperative of additional training for radiologists and the nuanced interpretation of biomarker data. The ongoing exploration of imaging biomarkers represents a dynamic frontier in the quest for more precise and personalized diagnostic tools, highlighting the research community's commitment to pushing the boundaries of medical imaging technologies.

New treatment strategies: At the forefront of ongoing research in acute pancreatitis is the quest for innovative treatment strategies encompassing realms such as immunomodulatory agents, stem cell therapy, and gene therapy. These pioneering approaches aim to revolutionize patient outcomes and mitigate the inherent complications associated with acute pancreatitis. The continuous exploration of novel treatment modalities reflects a commitment to pushing the boundaries of therapeutic interventions, aiming to improve the standard of care for patients grappling with acute pancreatitis. By fostering an environment of discovery and innovation, ongoing research endeavors promise to reshape the landscape of treatment options, ultimately enhancing the toolkit available to healthcare providers and improving the prognosis for individuals affected by acute pancreatitis and related conditions.

## Conclusions

The intricate landscape of enteral feeding strategies in acute pancreatitis reveals a dynamic interplay of conflicting evidence and evolving perspectives. EEF emerges as a promising approach, supported by numerous studies indicating positive outcomes such as reduced hospital stays, improved nutritional status, and a diminished risk of complications. However, controversies persist, as some studies challenge the universal benefits of early initiation, emphasizing potential risks in certain high-risk and severe cases. On the other hand, with its cautious approach, DEF gains traction in managing patients where the risks associated with early feeding are deemed higher. Identifying high-risk individuals and employing a severity-based approach become crucial in making informed decisions. Practical recommendations for clinicians emphasize an individualized approach, incorporating regular monitoring and adjustment of nutritional plans. As acute pancreatitis management continues to evolve, staying informed about emerging guidelines is paramount. This review aims to provide clinicians with a synthesized understanding of critical findings, equipping them to effectively navigate the complexities of enteral feeding decisions in acute pancreatitis.
